# Cold Press Pomegranate Seed Oil Attenuates Dietary-Obesity Induced Hepatic Steatosis and Fibrosis through Antioxidant and Mitochondrial Pathways in Obese Mice

**DOI:** 10.3390/ijms21155469

**Published:** 2020-07-31

**Authors:** Marco Raffaele, Maria Licari, Sherif Amin, Ragin Alex, Hsin-hsueh Shen, Shailendra P. Singh, Luca Vanella, Rita Rezzani, Francesca Bonomini, Stephen J. Peterson, David E. Stec, Nader G. Abraham

**Affiliations:** 1 Departments of Medicine and Pharmacology, New York Medical College, Valhalla, NY 10595, USA; marco.raffaele@hotmail.com (M.R.); marylicari89@gmail.com (M.L.); samin3@nymc.edu (S.A.); ralex@nymc.edu (R.A.); hshen2@nymc.edu (H.-h.S.); spbiotech2004@gmail.com (S.P.S.); 2 Department of Drug Sciences, University of Catania, 95123 Catania, Italy; lvanella@unict.it; 3 Departments of Biotechnology and Biomedical Engineering, Central University of Rajasthan, Rajasthan 305817, India; 4 Department of Clinical and Experimental Sciences, University of Brescia, 25121 Brescia, Italy; rita.rezzani@unibs.it (R.R.); francesca.bonomini@unibs.it (F.B.); 5 Department of Medicine, New York-Presbyterian Brooklyn Methodist Hospital, Brooklyn, NY 11215, USA; stp9039@nyp.org; 6 Department of Physiology and Biophysics, Cardiorenal and Metabolic Diseases Research Center, University of Mississippi Medical Center, Jackson, MS 39216, USA

**Keywords:** obesity, ROS, oxygen consumption, heme-oxygenase (HO-1), PGC-1α, insulin resistance

## Abstract

Aim: Obesity is associated with metabolic syndrome, hypertension, dyslipidemia, nonalcoholic fatty liver disease (NAFLD), and type 2 diabetes. In this study, we investigated whether the dietary supplementation of pomegranate seed oil (PSO) exerted a protective effect on liver lipid uptake, fibrosis, and mitochondrial function in a mouse model of obesity and insulin resistance. Method: In this in vivo study, eight-week-old C57BL/6J male mice were fed with a high fat diet (HFD) for 24 weeks and then were divided into three groups as follows: group (1) Lean; group (*n* = 6) (2) HF diet; group (*n* = 6) (3) HF diet treated with PSO (40 mL/kg food) (*n* = 6) for eight additional weeks starting at 24 weeks. Physiological parameters, lipid droplet accumulation, inflammatory biomarkers, antioxidant biomarkers, mitochondrial biogenesis, insulin sensitivity, and hepatic fibrosis were determined to examine whether PSO intervention prevents obesity-associated metabolic syndrome. Results: The PSO group displayed an increase in oxygen consumption, as well as a decrease in fasting glucose and blood pressure (*p* < 0.05) when compared to the HFD-fed mice group. PSO increased both the activity and expression of hepatic HO-1, downregulated inflammatory adipokines, and decreased hepatic fibrosis. PSO increased the levels of thermogenic genes, mitochondrial signaling, and lipid metabolism through increases in Mfn2, OPA-1, PRDM 16, and PGC1α. Furthermore, PSO upregulated obesity-mediated hepatic insulin receptor phosphorylation Tyr-^972^, p-IRB tyr^1146^, and pAMPK, thereby decreasing insulin resistance. Conclusions: These results indicated that PSO decreased obesity-mediated insulin resistance and the progression of hepatic fibrosis through an improved liver signaling, as manifested by increased insulin receptor phosphorylation and thermogenic genes. Furthermore, our findings indicate a potential therapeutic role for PSO in the prevention of obesity-associated NAFLD, NASH, and other metabolic disorders.

## 1. Introduction

According to the World Health Organization (WHO), obesity is defined as an abnormal or excessive accumulation of body fat that increases the risk of many health problems. In the United States, obesity affects 33% of the population, and the prevalence is expected to increase to 50% by 2030 [1]. Obesity is associated with an increased risk of metabolic syndrome, type 2 diabetes, hypertension, and cardiovascular disease [1,2,3]. The metabolic syndrome causes a spectrum of liver injury resulting in nonalcoholic fatty liver disease (NAFLD), which can progress to non-alcoholic steatohepatitis (NASH), fibrosis, and hepatocellular carcinoma [4,5]. Many pathways that are attributable to this complex pathophysiology are under investigation for drug development, with a focus on treating inflammation, as well as improving metabolic pathways and slowing or reversing liver fibrosis.

Recent studies have shown that oxidative stress and inflammation have been implicated in the progression of obesity and metabolic syndrome [6,7,8]. Oxidative stress is triggered by an imbalance between pro-oxidants such as reactive oxygen species (ROS), heme, and antioxidants like heme oxygenase (HO) [5,6,7]. Obesity produces oxidative stress through the increased generation of ROS, resulting in cytotoxicity [9,10,11]. Therefore, the upregulation of heme and ROS due to a decreased HO activity increases pre-adipocyte differentiation, as well as adipogenesis and the release of inflammatory adipokines such as nephroblastoma overexpressed gene (NOV), tumor necrosis factor-α (TNFα), and Interleukin-6 (IL-6) [11,12,13]. Interestingly, an increased ROS production does not increase HO-1 expression, leading to the resultant development of obesity and metabolic syndrome [10,11,14]. Recently, a novel adipokine, NOV, a member of the family of the cellular communication network (CCN), has been shown to be upregulated in HFD-fed mice [15,16]. The deletion of NOV improved glucose tolerance through an increase in mitochondrial biogenesis [16,17]. Conversely, the upregulation of NOV results in an increased adiposity, as well as increased plasma levels of cholesterol and triglycerides [15,16]. Obesity releases free fatty acids (FFA) that trigger the Toll-like receptor 4 (TLR4) signaling pathway, resulting in the activation of the nuclear factor-kappaB (NF-κB), a master transcription regulator in the production of pro-inflammatory cytokines, TNF-α [18,19,20]. TNF-α decreases insulin sensitivity, promotes powerful inflammatory mediators, specifically IL-6, and decreases anti-inflammatory cytokines such as adiponectin [19,20]. Thus, inflammatory adipokines such as NOV, TNF-α, and IL-6 have emerged as key regulators of obesity and insulin resistance. Matrix Metalloproteinase (MMP) is a group of enzymes that are zinc- and calcium-dependent endoproteinases that can degrade the extracellular matrix [21,22]. Obesity-associated liver inflammation and oxidative stress upregulate the expression of MMP-2 and MMP-9 [21,23]. MMP-2 and MMP-9 are known as gelatinase for their ability to degrade collagen type II and type IV, the major constituents of the basement membrane [23,24]. The destruction of the basement membrane results in dysfunction in the extracellular matrix, resulting in hepatic fibrosis [21,23]. Thus, the inhibition of MMP can have a potential therapeutic role in the progression of liver fibrosis. 

Recently, there has been growing interest in examining natural antioxidants found in dietary plants, which may have beneficial effects on metabolic disease. Previous studies have reported that curcumin, black seed oil, and resveratrol have beneficial effects on insulin sensitivity and on the inhibition of inflammation [25,26,27,28] Natural substances such as pomegranates may be beneficial towards combating obesity [29,30]. Pomegranate juice contains antioxidants such as polyphenols (0.2–1%), tannins, punicic acid, and ascorbic acid [31,32]. Although the essential oil extracted from pomegranate contains a rich fraction of these antioxidants and is superior to pomegranate juice, it also contains high levels of fructose (6.83/100 g) and glucose (6.66/100 g) [33]. It has been shown that fructose-induced obesity results in an inflammatory and oxidative state; therefore, it promotes the development of NAFLD and metabolic syndrome [34]. The potential adverse effects of high sugar-containing pomegranate juice was highlighted in a clinical trial that revealed that pomegranate juice administration over one month did not modify insulin secretion and sensitivity in patients with obesity [35]. PSO may exhibit higher therapeutic benefits for metabolic syndrome than those of pomegranate juice due to the lack of sugars and presence of enhanced antioxidants. Dietary PSO was shown to ameliorate high-fat diet-induced obesity and insulin resistance in mice, independent of changes in food intake or energy expenditure [36]. However, more research needs to be conducted in order to determine the beneficial effects of PSO in obesity-related metabolic syndrome and mitochondrial dysfunction because there are currently no conclusive studies that establish this association. Hence, the aim of this study was to determine the effects on mice fed an HFD that a dietary supplementation of 1% of PSO had on: body weight gain, hypertension, NAFLD, mitochondrial and thermogenic gene levels, and insulin sensitivity.

## 2. Results

### 2.1. Effects of PSO on Physiological Parameters

**Figure 1:**
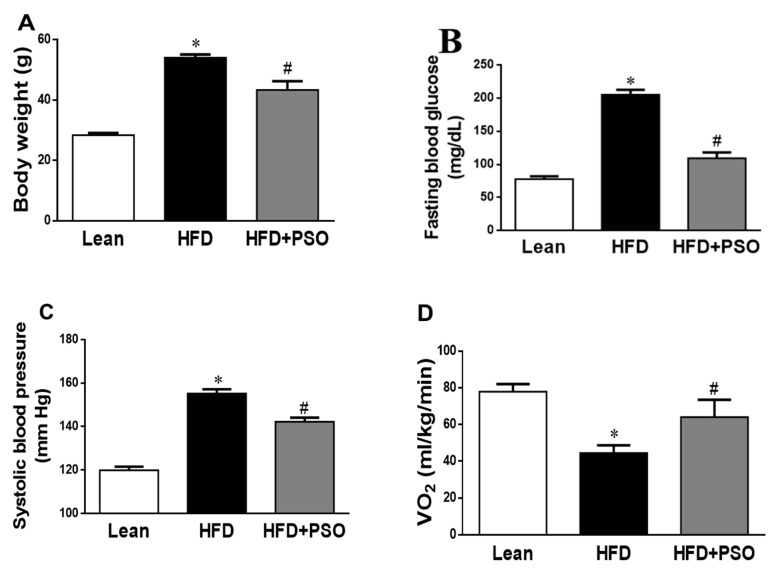
Effect of pomegranate seed oil (PSO) treatment on (**A**) Body weight, (**B**) Fasting blood glucose, (**C**) Systolic blood pressure, and (**D**) Oxygen consumption (VO_2_). * = *p* < 0.05 as compared to lean. # = *p* < 0.05 as compared to HFD. *n* = 6/group.

We analyzed the effect of PSO supplementation on physiological parameters in mice fed an HFD. As shown in Figure 1A, HFD feeding resulted in a significant increase in body weight as compared to lean mice (*p* < 0.05), and this increase in body weight was attenuated by PSO supplementation (50 ± 2 vs. 29 ± 1 vs. 42 ± 2 g: HFD, lean, HFD + PSO). Fasting blood glucose levels were significantly increased in HFD mice as compared to lean mice, and were significantly (*p* < 0.05) lowered by PSO (205 ± 5 vs. 78 ± 4 vs. 111 ± 2 mg/dL: HFD, lean, HFD + PSO) (Figure 1B). The blood pressure was also significantly (*p* < 0.05) increased in mice fed an HFD as compared to lean mice and PSO-supplemented mice (155 ± 2 vs. 120 ± 2 vs. 135 ± 5 mmHg: HFD, lean, HFD + PSO) (Figure 1C). Oxygen consumption was significantly (*p* < 0.05) decreased in mice fed an HFD (Figure 1D) and was returned to the levels observed in lean animals by PSO (45 ± 4 vs. 78 ± 4 vs. 61 ± 7 mL/kg/min: HFD, lean, HFD + PSO) (Figure 1D).

### 2.2. PSO Decreases Hepatic Steatosis and Fibrosis

**Figure 2:**
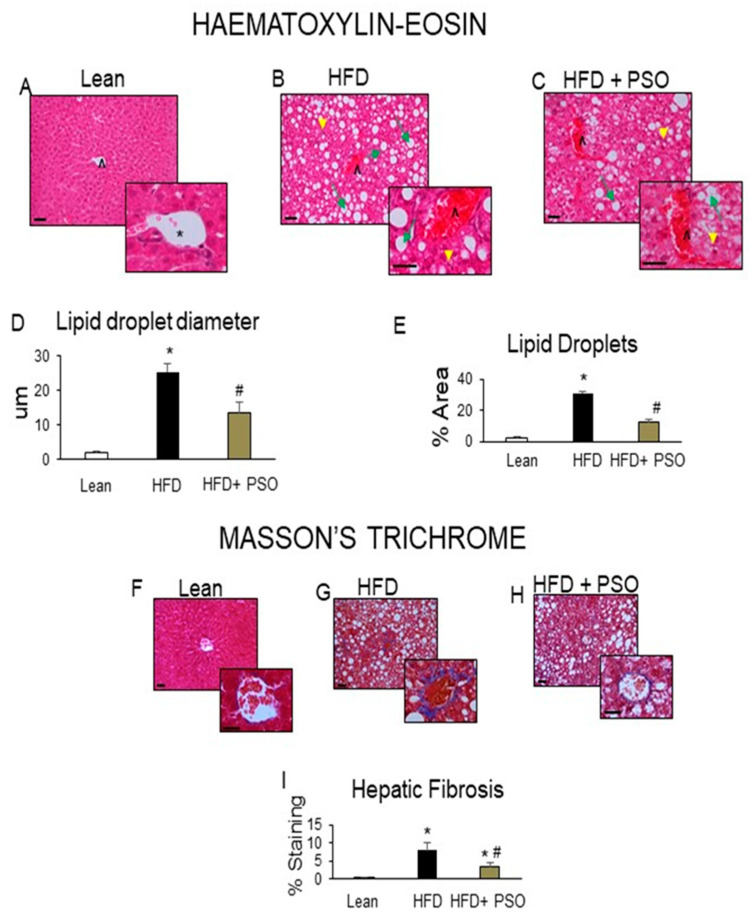
Histological analysis of livers. In lean mice, (**A**–**C**) Representative Haematoxylin-eosin (H&E) staining and (**D**,**E**) quantitation showed (**A**) a normal hepatic parenchyma with radially arranged rays of hepatocytes, regularly directed from the central vein of each lobule towards its periphery. (**B**) HFD-treated mice exhibited significant morphological alterations with lipid droplets deposition and inflammatory cells infiltration. (**C**) Hepatic parenchymal alterations in the HFD group were decreased in HF animals treated with PSO. (**D**) Quantitation of lipid droplet diameter. (**E**) Quantitation of the lipid droplet area. (**F**) Hepatic fibrosis by Masson’s staining in lean mice that exhibited very weak parenchymal fibrosis; (**G**) HFD mice demonstrating strong perisinusoidal collagen deposition; (**H**) HFD + PSO-treated mice demonstrating reduced perisinusoidal collagen deposition; (**I**) quantitation of collagen staining. * indicates a centrolobular vein. Green arrows indicate lipid droplets and yellow arrowheads indicate areas of lobular inflammatory loci. Bar = 50 µm. * *p* < 0.05 from corresponding value in lean mice. # *p* < 0.05 from corresponding value in HFD mice. *n* = 6/group.

Histological analyses of livers from lean mice revealed no significant steatosis, rare ballooning, no inflammatory foci, and no fibrosis (Figure 2A). The livers of HFD mice exhibited elevated steatosis (green arrows), moderate lobular inflammatory loci (yellow arrowheads), hepatocyte ballooning, and fibrosis (caret) (Figure 2B), which were all reduced by PSO treatment (Figure 2C). The morphometric analysis of liver lipid droplets showed that PSO treatment decreased the lipid droplet diameter when compared to the HFD group (*p* < 0.05) (Figure 2D). The lipid content was increased (*p* < 0.05) in the HFD group when compared to the lean group and was significantly (*p* < 0.05) reduced in PSO-treated mice (Figure 2E).

### 2.3. PSO Decreases Fibrotic Markers and Lowers Serum ALT and AST Levels in HFD-Fed Mice

**Figure 3:**
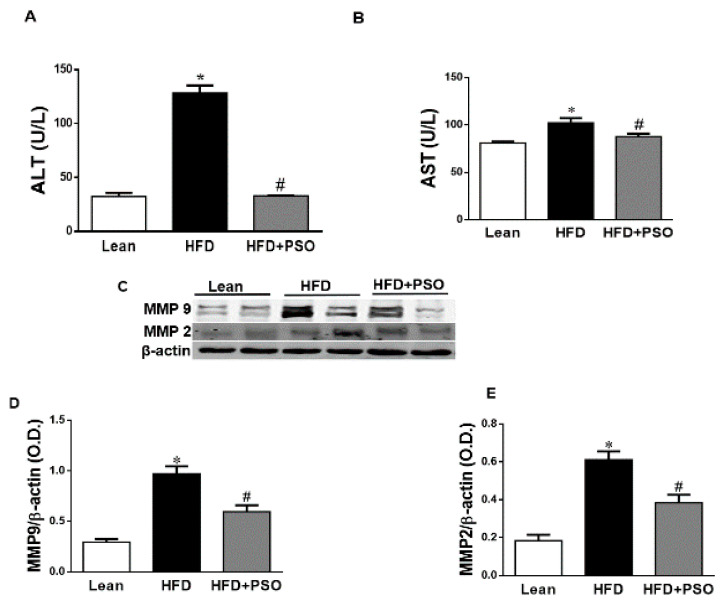
Serum alanine aminotransferase (*ALT*) and aspartate aminotransferase (*AST*) levels in lean, HFD-, and HFD + PSO-treated mice. (**A**) Serum ALT levels. (**B**) Serum AST levels. (**C**) Representative Western blot of hepatic MMP2 and MMP9 as well as β-actin. (**D**) Quantitation of the levels of hepatic MMP9 protein. (**E**) Quantitation of the levels of hepatic MMP2. * = different *p* < 0.05 from corresponding value in lean mice. # = different *p* < 0.05 from corresponding value in HFD mice. *n* = 6/group.

HFD increased hepatic collagen deposition, as measured by Masson’s Trichrome staining, when compared to lean mice (Figure 2F,G). PSO significantly (*p* < 0.05) decreased collagen deposition in mice fed an HFD (Figure 2G–I). Dietary-induced obese mice developed an impaired liver function as indicated by increased serum levels of AST (*p* < 0.05) and ALT (*p* < 0.05). PSO normalized the ALT and AST levels (*p* < 0.05) to the levels of the lean group (ALT, 128 ± 7 vs. 33 ± 4 vs. 33 ± 1 U/L: HFD, lean, HFD + PSO; AST, 99 ± 2 vs. 81 ± 2 vs. 87 ± 3: U/L HFD, lean, HFD + PSO) (Figure 3A,B). Fibrotic protein signaling was examined by measuring MMP2 and MMP9, which were significantly (*p* < 0.05) increased in the HFD group when compared to lean mice and PSO mice (MMP9, 1.1 ± 0.05 vs. 0.3 ± 0.04 vs. 0.6 ± 0.03 AU: HFD, lean, HFD + PSO; MMP2, 0.61 ± 0.04 vs. 0.18 ± 0.03 vs. 0.38 ± 0.04 AU: HFD, lean, HFD + PSO) (Figure 3C–E).

### 2.4. Effect PSO on Hepatic Inflammatory Proteins and Heme Oxygenase (HO)

**Figure 4:**
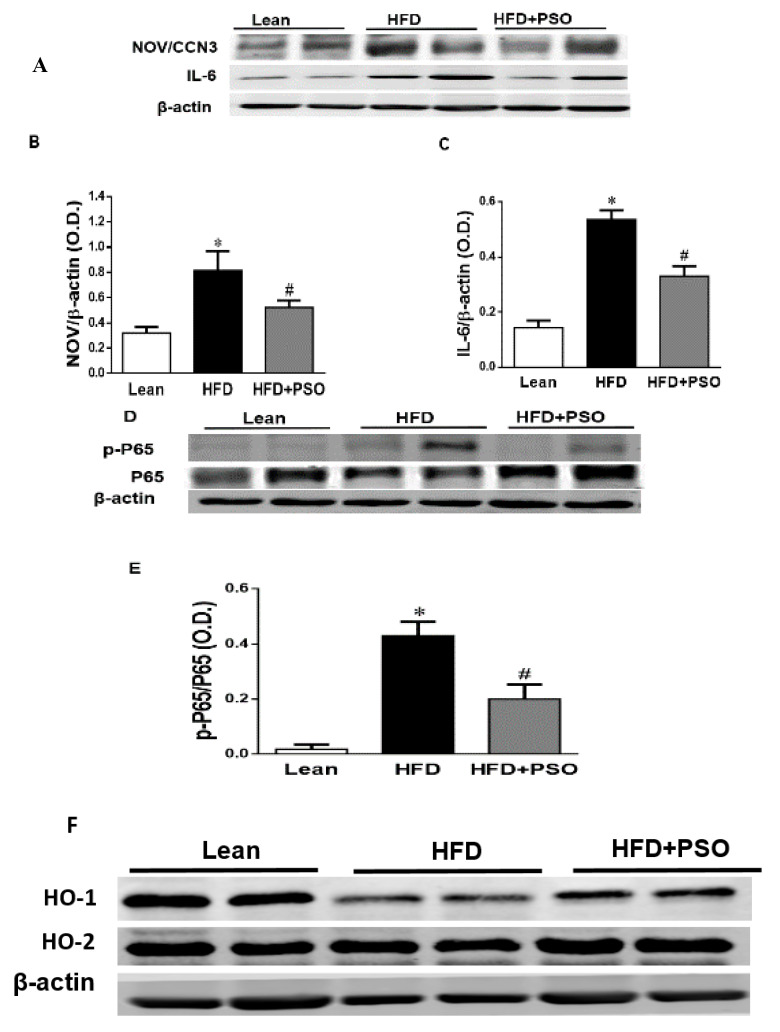

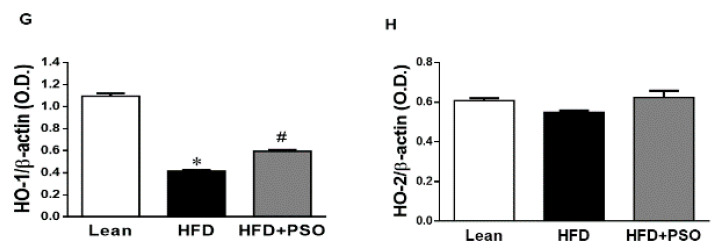
Hepatic inflammatory proteins and HO protein levels in lean, HFD-, and HFD+ PSO-treated mice. (**A**) Representative western blots for NOV/CCN3, interleukin-6 (IL-6), and β-actin. (**B**) Quantitation of the hepatic levels of NOV/CCN3 protein levels. (**C**) Quantitation of the hepatic levels of IL-6. (**D**) Representative western blots for p-P65, total P65, and β-actin. (**E**) Quantitation of the hepatic p-P65 to total P65 ratio. (**F**) Representative western blots for heme oxygenase-1 (HO-1), heme oxygenase-2 (HO-2), and β-actin. (**G**) Quantitation of the hepatic levels of HO-1. (**H**) Quantitation of the hepatic levels of HO-2. * = different *p* < 0.05 from corresponding value in lean mice. # = different *p* < 0.05 from corresponding value in HFD mice. *n* = 6/group.

The western blot analysis of the liver tissue of the HFD group displayed a significant increase (*p* < 0.05) in pro-inflammatory proteins, NOV, IL-6, and p-P65 levels, which were significantly decreased in PSO-supplemented mice (NOV, 0.82 ± 0.04 vs. 0.32 ± 0.04 vs. 0.52 ± 0.05 AU: HFD, lean, HFD + PSO; IL-6, 0.53 ± 0.03 vs. 0.14 ± 0.02 vs. 0.32 ± 0.03 AU: HFD, lean, HFD + PSO; p-P65, 0.43 ± 0.05 vs. 0.02 ± 0.01 vs. 0.19 ± 0.05 AU: HFD, lean, HFD + PSO) (Figure 4A–E). The levels of HO-1 were decreased in the livers of HFD mice, and (Figure 4F,G) PSO increased HO-1 protein levels (0.4 ± 0.01 vs. 1.1 ± 0.03 vs. 0.62 ± 0.01 AU: HFD, lean, HFD + PSO) (Figure 4F,G). HO-2 expression was not significantly affected by the treatments (0.55 ± 0.01 vs. 0.61 ± 0.01 vs. 0.61 ± 0.03 AU: HFD, lean, HFD + PSO) (Figure 4F–H).

### 2.5. Effect PSO on Mitochondrial Function and Lipid Metabolism

**Figure 5:**
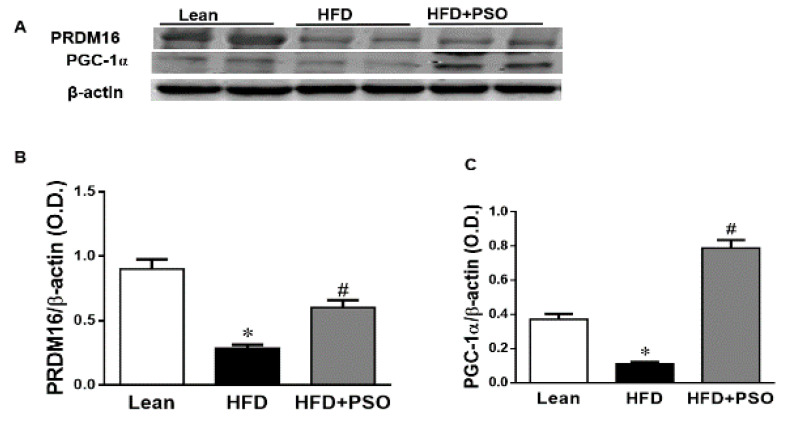

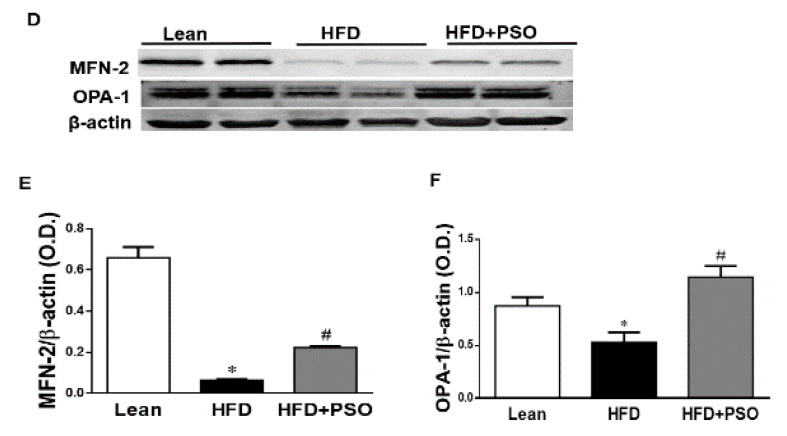
Hepatic levels of the PR domain containing 16 (PRDM16), peroxisome proliferator-activated receptor gamma coactivator 1-alpha (PGC-1α), Mitofusin-2 (MFN-2), and Dynamin-like 120 kDa protein (OPA-1) in lean, HFD-, and HFD + PSO-treated mice. (**A**) Representative western blots for PRDM16, PGC-1α, and β-actin. (**B**) Quantitation of the hepatic levels of PRDM16. (**C**) Quantitation of the hepatic levels of PGC-1α. (**D**) Representative western blots for MFN-2, OPA-1, and β-actin. (**E**) Quantitation of the hepatic levels of MFN-2. (**F**) Quantitation of the hepatic levels of OPA-1. * = different *p* < 0.05 from corresponding value in lean mice. # = different *p* < 0.05 from corresponding value in HFD mice. *n* = 6/group.

We measured the protein expression involved in the mitochondrial function and energy expenditure, including PRDM16, PGC-1α, MFN2, and Opa1. HFD markedly decreased (*p* < 0.05) the levels of these proteins, impairing the mitochondrial biogenesis, while treatment with PSO reversed these effects (PRDM16, 0.3 ± 0.03 vs. 0.91 ± 0.05 vs. 0.67 ± 0.04 AU: HFD, lean, HFD + PSO; PGC-1α, 0.11± 0.01 vs. 0.37 ± 0.03 vs. 0.78 ± 0.04 AU: HFD, lean, HFD + PSO; MFN2, 0.06 ± 0.01 vs. 0.65 ± 0.05 vs. 0.22 ± 0.01 AU: HFD, lean, HFD + PSO; Opa1, 0.53 ± 0.1 vs. 0.87 ± 0.08 vs. 1.14 ± 0.1 AU: HFD, lean, HFD + PSO) (Figure 5).

### 2.6. Effect PSO on Hepatic Insulin Resistance

**Figure 6:**
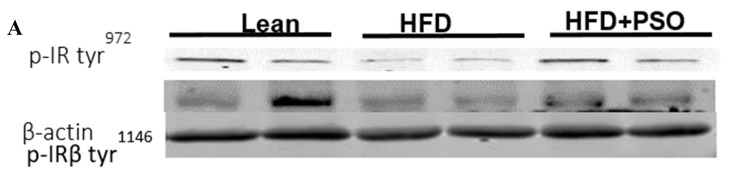

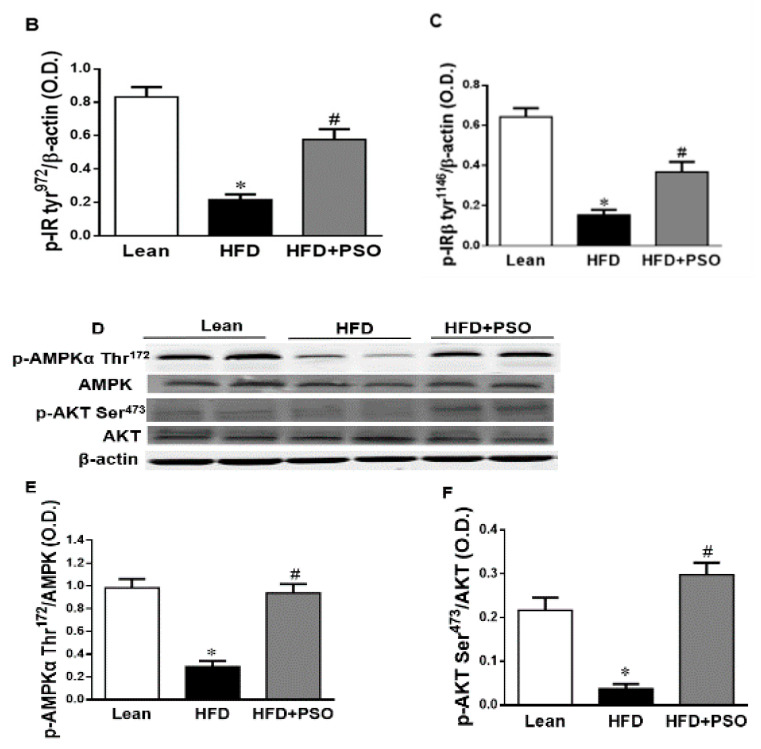
Hepatic levels of phosphorylated insulin receptor tyrosine 972 (pIRTyr972), phosphorylated insulin receptor-β tyrosine 1146 (pIRβTyr1146), total 5’ AMP-activated protein kinase (AMPK), phosphorylated AMPK, total Akt, and phosphorylated Akt in lean, HFD-, and HFD + PSO-treated mice. (**A**) Representative western blots for pIRTyr972, pIRβTyr114, and β-actin. (**B**) Quantitation of the hepatic levels of pIRTyr972. (**C**) Quantitation of the hepatic levels of pIRβTyr114. (**D**) Representative western blots for pAMPK, total AMPK, pAkt, total Akt, and β-actin. (**E**) Quantitation of the hepatic levels of pAMPK/total AMPK. (**F**) Quantitation of the hepatic levels of pAkt/total Akt. * = different *p* < 0.05 from corresponding value in lean mice. # = different *p* < 0.05 from corresponding value in HFD mice. *n* = 6/group.

HFD mice exhibited lower hepatic levels of proteins regulating glucose homeostasis, e.g., the insulin receptors pIR-tyr972, pIR-tyr1146 and pAMPK, pAKT, when compared to lean mice, which was reversed in PSO-supplemented mice (pIRtyr972, 0.21 ± 0.03 vs. 1.62 ± 0.16 vs. 0.47 ± 0.03 AU: HFD, lean, HFD + PSO; pIRtyr1146, 0.15 ± 0.03 vs. 0.64 ± 0.04 vs. 0.36 ± 0.05 AU: HFD, lean, HFD + PSO; pAMPK, 0.29 ± 0.04 vs. 0.98 ± 0.07 vs. 0.93 ± 0.07 AU: HFD, lean, HFD + PSO; pAKT, 0.03 ± 0.005 vs. 0.22 ± 0.02 vs. 0.29 ± 0.03 AU: HFD, lean, HFD + PSO (Figure 6).

## 3. Discussions

In this study, we demonstrate the beneficial effects of PSO supplementation on physiological parameters such as blood pressure, fasting blood glucose, and body weight. Additionally, PSO supplementation in mice consuming an HFD increased oxygen consumption to the levels of the lean animals, indicating an improvement in the mitochondrial burning of fatty acids (Figure 1). 

Supplementation of obese mice with PSO decreased hepatic steatosis and fibrosis. PSO decreased the lipid droplet diameter when compared with the liver of HFD mice, which showed elevated steatosis, hepatocyte ballooning, and fibrosis (Figure 2). Overall, the lipid content of the liver in mice supplemented with PSO was lowered. This resulted in improving the liver function, as indicated by decreased serum levels of AST and ALT, which are markers of liver dysfunction (Figure 3A,B). The degradation of the extracellular matrix is a key factor in the development and progression of hepatic fibrosis. This process is regulated by matrix metallopeptidases (MMPS) such as MMP2 and 9. An HFD causes the remodeling of MMP 2 and 9, which are precursors of hepatic fibrosis, followed by cirrhosis, liver failure, and hepatocellular carcinoma. In the present study, an HFD increased the hepatic levels of MMPS 2 and 9 and induced hepatic fibrosis (Figure 2). PSO supplementation resulted in a decrease in both MMP2 and 9, as well as in hepatic collagen staining (Figure 3C–E). These results highlight the anti-fibrotic potential of PSO and suggest that supplementation of PSO may be an effective measure against the progression of NAFLD to NASH.

Dietary supplements such as PSO play a significant role in promoting weight loss, as well as having a direct effect on the liver by decreasing hepatic steatosis. PSO is a highly rich source of polyphenols, e.g., ellagitannins, anthocyanin, ascorbic acid, and punicic acid, known for their potent antioxidant capacity. Although pomegranate juice is an excellent source of these antioxidants, it contains fructose and glucose, which may not be suitable for obese patients, diabetic patients, and patients with NAFLD. Additionally, a recent human study showed that pomegranate juice supplementation lowered the level of systolic and diastolic blood pressure in patients with metabolic syndrome, but there was no significant difference in fasting blood glucose, insulin, and HOMA-IR [37]. These results suggest that the high levels of sugars found in pomegranate juice may attenuate its protective effects on insulin resistance. These limitations are not found in PSO, as this extract does not contain the high levels of sugars found in pomegranate juice.

Non-alcoholic fatty liver disease (NAFLD) affects ~25% of adults and is the most common cause of chronic liver disease in the western world [38,39]. It is related to metabolic syndrome, i.e., obesity, hyperlipidemia, type 2 diabetes, hypertension, and associated cardiovascular disease, which serves as a marker of increased morbidity and mortality [40,41]. Dietary-induced obesity results in hepatic fat accumulation, fatty liver disease, and deterioration in the liver function. There are currently no specifically approved drugs for the treatment of NAFLD. The primary treatment of NAFLD consists of lifestyle modifications designed to result in weight loss [42,43,44].

One of the pathways most impacted by dietary-induced obesity is the inflammatory pathway. Hepatic inflammation and oxidative stress result in mitochondrial damage and changes in mitochondrial dynamics [3,45]. This manifests as hepatocellular oxidative damage, resulting in hepatic inflammation (non-alcoholic steatohepatitis). An HFD enhances FFA generation and increases mitochondrial dysfunction and ROS levels [46]. Mitochondrial dysfunction results in decreased beta-oxidation in the liver, allowing fat to accumulate, resulting in a “Fatty Liver” [47,48,49]. Peroxisome proliferator-activated receptor-gamma coactivator (PGC)-1 alpha is a member of the family of transcription coactivators that plays a central role in the mitochondrial biogenesis adaptive thermogenesis program, including the stimulation of energy uptake and mitochondrial fatty acid oxidation [9,50,51]. Previous studies have shown that the downregulation of PGC-1α leads to mitochondrial dysfunction, as manifested by a simultaneous reduction in mitochondrial fusion protein mitofusin 2 (Mfn-2) and thermogenic genes such as the PR domain containing 16 (PRDM16). Reductions in these genes are associated with obesity and insulin resistance [9,51,52]. Mitochondrial network dynamics are tightly linked to energy and metabolic demands, as well as to mitochondrial quality control (MQC). Mitochondrial network dynamics are regulated by fission and fusion proteins [53,54,55,56]. Mitochondrial fusion is facilitated by Mfn-2 on the mitochondrial outer membrane and by optic atrophy 1 protein (OPA1), whereas mitochondrial fission proteins are primarily regulated by GTPase dynamin-related protein 1 (Drp1) [53,54,55,56]. Upon stimulation, mitochondria undergo a consistent interchange to either a more filamentous or more fragmented state through fission or fusion processes in order to adapt the mitochondrial function to actual energetic and metabolic demands [53,54]. Previous studies have shown that the deletion of Mfn-2 and OPA-1 in brown adipose tissue remodels the mitochondrial dysfunction, causing a decrease in insulin resistance and energy expenditure [9,55,56]. These inflammatory and metabolic pathways are involved in obesity-related metabolic syndrome and mitochondrial dysfunction [3,57]. Supplementation of dietary-induced, obese mice with PSO improved both the mitochondrial function and dynamics in hepatic steatosis by decreasing the levels of inflammatory markers, NOV, IL-6, and phosphorylated P65, which is a major regulator of IKKβ/NF-KB signaling (Figure 4A–E). This results in increased mitochondrial dynamics (fusion/fission), biogenesis, and function, leading to decreased oxidative stress and inflammation and the activation of anti-apoptotic genes (p65) in obese mice. 

PSO is rich in polyphenols that are natural antioxidants and that can further act to decrease hepatic oxidative stress to improve hepatic lipid metabolism and hepatic insulin resistance. One of the most important findings of the present study was the induction of HO-1 by PSO supplementation (Figure 4F–H). The HO-1 pathway plays an important role in the regulation of body weight, adipose tissue expansion, insulin resistance, and hepatic steatosis [58,59,60,61,62]. HO-1 interacts with various pathways, such as sirtuin-1 (SIRT1) and PGC1α, to improve cell function in obesity [9,63,64]. PSO supplementation not only increased the hepatic HO-1 level but also the levels of PGC1α, demonstrating the close link between these two pathways (Figure 5A–C). Furthermore, HFD mice supplemented with PSO exhibited an increased expression of the genes regulating mitochondrial dynamics, such as PRDM16, PGC1α, MFN2, and OPA-1, as compared to HFD alone (Figure 5D–F). PSO supplementation attenuated the mitochondrial fission potential and improved mitochondrial fusion-associated proteins in mice fed an HFD.

Our earlier studies demonstrated an increased NOV expression in adipose tissue in obese mice when compared to lean mice [16], and in the current study the hepatic NOV expression in HFD-obese mice supplemented with PSO was lower than in mice that were fed HFD alone. Enhanced levels of NOV are associated with increased hepatic levels of inflammatory cytokines, which deleteriously affect insulin signaling, resulting in hepatic insulin resistance [15]. In contrast, the downregulation of NOV results in a decrease in both adipose tissue accumulation and inflammatory cytokines, as well as an increased insulin sensitivity in PSO-supplemented HFD mice [56]. As a result, PSO supplementation resulted in the attenuation of several inflammatory mediators in the liver and improved the overall liver function in HFD-fed mice. 

**Figure 7:**
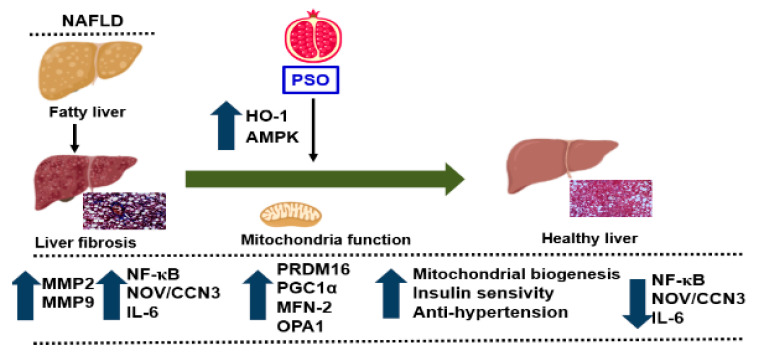
Schematic depiction of PSO effects on fatty liver. PSO attenuates obesity-induced hepatic steatosis and fibrosis through an increase in the antioxidant gene HO-1 and the mitochondrial signaling pathways PRDM 16, PGC1α, MFN-2, and OPA1. Additionally, there is a decrease in the fibrotic markers MMP2, MMP9 and inflammatory adipokines NF-κB, Nov/CCN3, and IL-6, resulting in a healthy liver.

PSO improved hepatic insulin resistance and increased insulin receptor phosphorylation via increased proteins regulating glucose homeostasis, e.g., p-AMPK, p-AKT, p-IR-tyr972, and p-IR-tyr1146 (Figure 6A–F). All of these pathways play an important role in liver insulin signaling, and all are affected by dietary-induced obesity [65,66]. Figure 7 shows that an HFD increases lipid deposit and fibrosis, which is associated with increases in MMP 2, MMP9, NF-_K_B, NOV/CCN3, and IL6. PSO supplementation increases antioxidants gene HO-1, which is associated with an increase in the mitochondrial fusion protein MFn2, OPA1, PGC1α, and the thermogenic proteins PRMD 16. PSO-mediated increases in mitochondrial biogenesis are associated with increases in oxygen consumption rates and a lower blood pressure in obese mice.

There are several limitations with the current study, which need to be considered prior to the results of this study being translated for humans. First, the duration of the PSO supplementation in the current study was only eight weeks. It is possible that a more beneficial action of PSO supplementation could be achieved with a longer supplementation time frame. Second, the dose of PSO utilized in the present study could be increased to provide additional benefits. PSO contains polyphenols and punicic acid, which are also found in other dietary supplements. Thus, future studies comparing the effectiveness of PSO to other dietary supplements such as curcumin, black seed oil, and resveratrol are needed in order to determine the relative efficacy of each of these supplements in attenuating markers of metabolic syndrome.

## 4. Materials and Methods

### 4.1. Animal Protocols and Measurement of Body Weight

**Figure 8:**
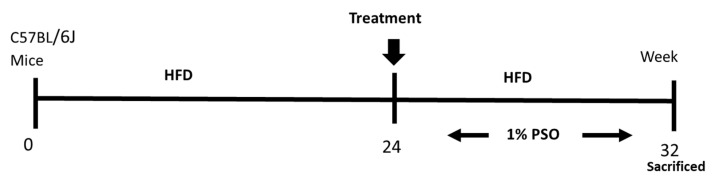
Schematic depiction of experimental protocol. C57BL/6J mice were fed a high fat diet (HFD) for 24 weeks after which time they were randomly assigned to a treatment group in which they were supplemented to pomegranate seed oil (PSO) for an additional 8 weeks. Mice were then euthanized at 32 weeks on the HFD.

Eight-week-old C57BL/6J male mice were divided into three treatment groups of six animals as follows: group (1) Lean; group (2) HFD; group (3) HFD treated for the last eight weeks with PSO at a concentration of 40 mL per kg of food PSO (HFD + PSO group) (Figure 8). This concentration of PSO is equivalent to 160–200 mg PSO consumed per day, given that the mice consume between 4–5 g of chow per day. The HFD group and the HFD and PSO group were fed western diets with a 51% fat content, while lean mice were provided regular diets (Harlan, Teklad Lab animal diets, Indianapolis, IN, USA) for 24 weeks. For the additional eight weeks, the HFD in the PSO group was supplemented with 1% PSO (vol/wt). The formulation of PSO used was as follows; 1% PSO, p-Cymene 1.24%, Carvacrol 0.08%, FFA 1, 29%, Oleic Acid 21.53%, palmitic acid 11.31%, linoleic acid 57.44%, and other fatty acid 1.98%, (Trinutra Ltd., www.trirnutra.Com, Hamazmera 9, Nes Ziona, Israel). 

The PSO was mixed into the HFD food, made into pellets using a mixer, and given for eight weeks, as shown in the scheme. After eight weeks, the mice were euthanized. All animal experiments were performed at the New York Medical College and followed the New York Medical College IACUC institutionally approved protocol, in accordance with NIH guidelines (Protocol # 22-2-0415H, 18 April 2018). Mice were euthanized with ketamine (100 mg/kg)/xylazine (10 mg/kg) injection, followed by cervical dislocation and tissue collection.

### 4.2. Determination of Blood Glucose, Blood Pressure, and VO_2_

Fasting BG was measured from tail blood following a six-hour fast. BP was measured via the tail-cuff method using the CODA tail-cuff System (Kent Scientific, Torrington, CT, USA). Mice were acclimated to the oxygen consumption chamber over a three-week period in two-hour increments, three times a week. The Oxylet gas analyzer and airflow unit (Oxylet, Panlab-Bioseb, Vitrolles, France) were used to determine mouse VO_2_. Each mouse was placed individually in the instrument, and VO_2_ and VCO_2_ were measured. The observational data for VO_2_ are expressed as the consumed oxygen per kilogram BW per minute (ml/kg/min) [3,56]. The respiratory quotient (RQ) was calculated as VCO_2_/VO_2._

### 4.3. Western Blot Analysis and Histological Evaluation

For protein expression analyses, liver tissues were lysed in RIPA lysis buffer supplemented with protease and phosphatase inhibitors (CompleteTM Mini and PhosSTOPTM, Roche Diagnostics, Indianapolis, IN, USA). Frozen mouse adipose tissue was ground under liquid nitrogen and suspended in homogenization buffer (comprising mmol/L: 10 phosphate buffer, 250 sucrose, 1.0 EDTA, 0.1 PMSF, and 0.1% *v*/*v* tergitol, pH 7.5). For the western blot analysis, pelleted cells were lysed, and primary antibodies of MMP9, MMP2, NOV/CCN3, IL-6, pP65, P65, PRDM16, MFN2, OPA1, pAMPK, AMPK, pAKT, AKT, pIR tyr1146, β-actin (all cell signaling technology, Danvers, MA, USA), HO-1, HO-2 (Enzo Life Sciences, Farmingdale, NY, USA), PGC-1α (Santa Cruz Biotechnology, Santa Cruz, CA, USA), and pIR tyr972 (Millipore, Bedford, MA, USA) proteins were incubated. Protein detection was carried out using a secondary infrared fluorescent dye conjugated antibody absorbing at both 800 nm and 700 nm. The blots were visualized using an Odyssey Infrared Imaging Scanner (Li-Cor Science Tec) and quantified by densitometric analysis performed after normalization with β-actin. The results were expressed as arbitrary units (AU).

Liver samples from each experimental group were fixed in 4% paraformaldehyde, dehydrated, embedded in paraffin wax, and sectioned (6 μm thick). The sections were deparaffinized, rehydrated, and stained with haematoxylin-eosin (H&E) and Masson’s trichrome staining. The main liver histopathological features were described, including steatosis, inflammation, and fibrosis. Steatosis was assessed on hematoxylin-eosin stained sections by counting 200 randomly chosen lipid droplets/groups and selecting their diameter (μm) and volume density (%) at a final magnification of 400×. Fibrosis was evaluated and measured as a percentage of Masson’s blue staining at a final magnification of 400× [67,68].

The morphometrical analyses were performed by two different observers blinded to the experimental group using computer image analysis software and were then analyzed using an image program (Image Pro Premier 9.1, Media Cybernetics Inc., Rockville, MD, USA), performed as previously described [67,68].

### 4.4. Measurement of Serum Aspartate Aminotransferase (AST) and (ALT)

The serum concentrations of aspartate aminotransaminase (AST) and alanine aminotransferase (ALT) were evaluated by an enzyme-linked immunosorbent assay (ELISA) kit (Abcam, Cambridge, MA, USA) according to the manufacturer’s instructions. Briefly, 900 μL of reagent was incubated for 3 min at 37 °C, followed by adding 45 μL serum or blank (45 μL dd H_2_O). After incubating for 10 min, the absorbance at 450 nm was measured using a spectrophotometer (Synergy HT, BioTek Instrument Inc., Winooski, VT, USA).

### 4.5. Statistical Analysis

Data are expressed as means ± S.E.M. Bonferroni’s post-test analysis for multiple comparisons was used to calculate the significance of mean value differences using a one-way analysis of variance. The null hypothesis was rejected at *p* ˂ 0.05.

## 5. Summary

The obesity epidemic is the major driving force behind the increasing incidence of NAFLD and its resulting complications, including liver cirrhosis and liver cancers. Currently, there are no FDA approved drugs for the specific treatment of NAFLD. Lifestyle modification with the specific goal of weight loss is the first line of treatment for NAFLD. The results of the present study demonstrate that dietary supplementation with PSO could provide an additional defense against the development and progression of NAFLD and its associated cardiovascular morbidities. Our current study shows that obesity-induced hepatic steatosis and fibrosis that end in NAFLD are markedly attenuated by dietary PSO supplementation. PSO supplementation results in decreased levels of adipokines and inflammatory markers, e.g., levels of NF-_K_B, NOV/CCN3, IL-6, MMP2, and MMP9. PSO also contains antioxidants and increases the levels of HO-1, which has a positive effect on mitochondrial function and increases mitochondrial signaling by increasing PRDM16, PGC1α, MFN-2, and OPA1. As a result, mitochondrial biogenesis increases and insulin sensitivity increases, resulting in a healthy liver (Figure 7).

## Abbreviations

PSO	pomegranate seed oil
HO-1	heme oxygenase-1
FA	fatty acid
FFA	free fatty acid
FAS	fatty acid synthase
FGF21	fibroblast growth factor 21
HF	high fat
HFD	high fat diet
IR	insulin resistance
DM	diabetes mellitus
PGC-1α	peroxisome proliferator-activated receptor gamma coactivator 1-α
SIRT3	sirtuin3
DRP1	dynamin-related protein 1
Fis1	mitochondrial fission 1protein
OPA1	optic atrophy 1 protein
Mfn 1	mitochondrial fusion protein mitofusin 1
Mfn 2	mitochondrial fusion protein mitofusin 2
UCP1	uncoupling protein 1
MnSOD	mitochondrial superoxide dismutase
PRDM16	PR domain containing 16
VO_2_	oxygen consumption
Drp1	dynamin-related protein 1
ROS	reactive oxygen species
ALT	alanine aminotransferase
AST	asparagine aminotransferase
MMP	matrix metalloproteinase
